# Cytokines and immune biomarkers in neurodegeneration and cognitive function: A systematic review among individuals of African ancestry

**DOI:** 10.1002/alz.70514

**Published:** 2025-07-25

**Authors:** Maxwell Hubert Antwi, Ansumana Bockarie, George Nkrumah Osei, David Mawutor Donkor, David Larbi Simpong

**Affiliations:** ^1^ Department of Medical Laboratory Science School of Allied Health Sciences University of Cape Coast Cape Coast Ghana; ^2^ Department of Medical Laboratory Science Faculty of Health Sciences Koforidua Technical University Koforidua Ghana; ^3^ Department of Internal Medicine and Therapeutics School of Medical Sciences University of Cape Coast Cape Coast Ghana

**Keywords:** African ancestry, cognition, cytokines, immune markers, neurodegeneration

## Abstract

**BACKGROUND:**

Cytokines and immune markers are critical in mediating inflammation associated with neurodegenerative disease. This review analyzes the role of inflammatory cytokines and immune markers in neurodegeneration among African populations.

**METHODS:**

Following Preferred Reporting Items for Systematic Reviews and Meta‐Analyses guidelines, we conducted a systematic review of studies examining cytokine levels in neurodegenerative diseases, focusing on populations of African ancestry.

**RESULTS:**

Cytokines like interleukin (IL)‐6, soluble tumor necrosis factor receptor 1, plasma brain‐derived neurotrophic factor, and IL‐8. IL‐6 emerged as a key pro‐inflammatory marker linked to neurodegenerative diseases and cognitive decline, showing stronger correlations in African ancestry populations compared to Caucasians. Genetic findings revealed triggering receptor expressed on myeloid cells 2 variants and Fc fragment of IgG receptor IIb rs1050501 genotypes as influential in Alzheimer's disease (AD)‐related inflammation, alongside a unique correlation between immunoglobulin G index inflammatory markers and AD in African ancestry populations.

**DISCUSSION:**

The findings emphasize the crucial role of cytokines in the pathophysiology of neurodegenerative diseases. Understanding their variations among African populations can inform targeted therapeutic strategies and improve patient outcomes.

**Highlights:**

- Interleukin (IL)‐6 was identified as a key pro‐inflammatory marker, consistently linked to Alzheimer's disease (AD), Parkinson's disease, and dementia, underscoring its significant role in neurodegenerative disease progression.
- Brain‐derived neurotrophic factor levels were associated with improved cognitive performance, particularly in African American participants.
- Observational studies identified sex‐based differences in IL‐10 levels, particularly among older African American women.
- The review highlights notable ethnic differences in cytokines like IL‐8, IL‐1β, and soluble tumor necrosis factor receptors, emphasizing their roles in neurodegeneration and cognitive decline in people of African descent.
- Triggering receptor expressed on myeloid cells 2 variants, immunoglobulin GM allotypes, and Fc fragment of IgG receptor IIb rs1050501 genotypes were found to influence AD‐related inflammation and progression in African ancestry populations.

## 1 BACKGROUND

Neurodegenerative diseases, characterized by progressive loss of neurons and cognitive decline, affect millions of people worldwide and pose significant challenges for diagnosis and management.^1^, ^2^ Cytokines and immune markers act as signaling molecules that mediate and regulate immunity, inflammation, and hematopoiesis.^3^ In recent years, there has been growing interest in understanding the role of these biomarkers in various diseases, particularly multiple sclerosis (MS), cognitive impairments, and neurodegenerative disorders such as Alzheimer's disease (AD) and Parkinson's disease (PD).^4^ The intricate interplay among cytokines, immune markers, and neurodegenerative disease progression highlights the importance of studying these factors in diverse populations to develop targeted interventions and therapies. Cytokines, such as interleukins (ILs), tumor necrosis factor alpha (TNF‐α), and C‐reactive protein (CRP), have been implicated in the pathophysiology of numerous diseases.^5^ Their levels can indicate the presence and severity of inflammation, which is a common underlying mechanism in many chronic conditions and neurodegeneration.

Studies have correlated inflammatory markers with cognitive impairment and neurodegeneration among ethnic or racial groups.^6^ There are inconsistent clinical data for individual cytokines across many studies done outside the African continent and in those of African ancestry. A meta‐analysis that was published in 2010 including 40 studies reported that AD patients had increased circulating blood levels for TNF‐α, transforming growth factor beta (TGF‐β), IL 1 beta (IL‐1β), IL‐6, IL‐12, and IL‐18.^7^ The study, however, demonstrated no significant differences between AD patients and controls with respect to circulating blood levels of IL‐4, IL‐8, and IL‐10.^7^ Another meta‐analysis, still outside the context of the African region, showed significant associations between circulating blood levels of IL‐1β, IL‐2, IL‐10, TNF, IL‐6, and PD patients.^8^ With respect to amyotrophic lateral sclerosis (ALS), significantly higher blood levels of IL‐1β, IL‐6, IL‐8, TNF‐α, TNF receptor 1 (TNFR1), and vascular endothelial growth factor (VEGF) were found in the disease condition compared to the control group.^9^ These meta‐analyses have clarified the circulating blood cytokine levels in ALS, AD, and PD with not too high sample sizes and provided potential biomarkers for these neurodegenerative diseases.

Significant and aberrant cytokine levels in cerebrospinal fluid (CSF) of AD, PD, and ALS patients have been reported apart from those that have been reported in peripheral blood.^10^ In addition to immune markers and their association with neurodegeneration and cognition, other molecules like brain‐derived neurotrophic factor (BDNF), genetic markers such as the triggering receptor expressed on myeloid cells 2 (*TREM2*) gene, immunoglobulin G (IgG) index genes, α1‐antichymotrypsin (α1‐ACT), monokine induced by gamma inteferon (MIG), tumor necrosis factor–related apoptosis‐inducing ligand (TRAIL), Fas‐associated death domain (FADD), C‐C motif chemokine ligand 25 (CCL25), CCL26, and fractalkine (CX3CL1) have also been associated with an increased risk of AD.^11^ BDNF has emerged as a key regulator of neuronal survival, synaptic plasticity, and memory formation, highlighting its neuroprotective role. Similarly, genetic markers such as TREM2 have been recognized for their influence on microglial function, brain homeostasis, and inflammatory modulation in neurodegeneration and injury. The IgG index in CSF also serves as a valuable indicator of intrathecal IgG synthesis, reflecting inflammation or autoimmune activity within the central nervous system, particularly in MS. Additionally, ACT, an acute phase glycoprotein, plays a crucial role in amyloid beta (Aβ) polymerization and plaque formation, solidifying its association with AD. These markers provide valuable insights into the biological processes underlying the disease and can help identify individuals at higher risk.^12^

Whereas most studies have reported on some levels of protein misfolding (tau and tangles) and their genetic variation as pathognomonic biomarkers for neurodegenerative diseases, pro‐ and anti‐inflammatory cytokine levels and immune markers correlation for early detection of these disorders in the African context and among the Black are not well understood.^13^ Moreover, the specific immune markers and cytokine responses in African populations in enhancing early detection of these neurodegenerative disease and their intervention strategies have been underexplored.

Despite advancements in management, treatment modalities, and diagnostic platforms for these debilitating conditions, the burden of neurodegeneration and cognitive impairment continues to rise.^1^, ^2^ Current diagnostic platforms predominantly rely on genetic and immune markers derived from Western‐centric reference datasets, limiting their applicability to African populations, who remain significantly underrepresented in biomarker and inflammatory studies.^13^ Emerging evidence suggests that individuals of African ancestry may exhibit distinct disease progression trajectories in AD, PD, and MS.^14^ Notably, the influence of apolipoprotein E (*APOE*) ε4 on AD risk, extensively documented in Western populations, appears to be less pronounced in older sub‐Saharan Africans.^15^ This underscores the critical need to delineate cytokines and immune markers relevant to neurodegenerative diseases in African populations and individuals of African descent.

To the best of our knowledge, this review is the first work to analyze data and findings from different studies examining the association between cytokine and immune marker levels and cognitive functions, inflammatory genetic predispositions, and neurodegeneration across diverse African populations and ancestry. This systematic review identifies key cytokines, immune markers, and related molecules and genetic markers associated with neurodegenerative diseases and cognitive impairment in African populations and those of African ancestry. The findings will enhance our understanding of the underlying biological mechanisms and inform future research in this area. Investigating the inflammatory processes involved in neurodegeneration and cognitive function may not only enhance the understanding of their pathophysiology but also pave the way for novel diagnostic and therapeutic strategies aimed at mitigating cognitive decline and neurodegenerative disorders.^16^

## 2 METHODS

The review protocol was developed in accordance with the recommended guidelines of PRISMA (Preferred Reporting Items for Systematic Reviews and Meta‐Analyses). Prior to the initiation of search, the protocol was registered on PROSPERO (registration ID:CRD420251004291).^17^ A PICO (population, intervention, comparison, outcome) tool was used to select the components of the review question. The following were applied: studies involving cytokines, chemokines, or inflammation and neurodegenerative diseases, dementia, or cognitive impairment in Africa and African ancestry without any restriction to age or sex. Study designs that qualified for this review included observational studies (cohort, case–control, and cross‐sectional), studies reporting on inflammatory biomarker (cytokine or immune marker) association with neurodegenerative diseases (dementia or cognitive impairment). Studies involving participants who are not of African ancestry or not published in English were excluded. African ancestry was defined based on the populations included in the reviewed articles, which encompassed individuals from African populations and ancestry. In addition, these classifications took into account geographical origins and self‐reported ancestry as specified in the respective studies. Again, studies involving non‐inflammatory (cytokines or immune markers) biomarkers with neurodegenerative disease (dementia or cognitive impairment) were excluded. Language barrier impedance for accurate interpretation and critical appraisal of studies, and limited resources for translating non‐English texts were some of the reasons for excluding studies published in non‐English languages. An extensive literature search was performed in PubMed and Google Scholar, covering studies from the origin of each database to February 2025. Relevant articles were identified using keywords and Boolean operators, and the search was further supplemented by reviewing gray literature. Searching government reports, conference proceedings, theses, and unpublished studies beyond traditional academic databases in ensuring wider and detailed coverage of available information for this review was used in the gray literature review. Google Scholar and specific institutional repositories were used for the gray literature to ensure that findings were not reduced to availability of research documents in conventional academic sources, and also not limited by publication bias.

**FIGURE 1:**
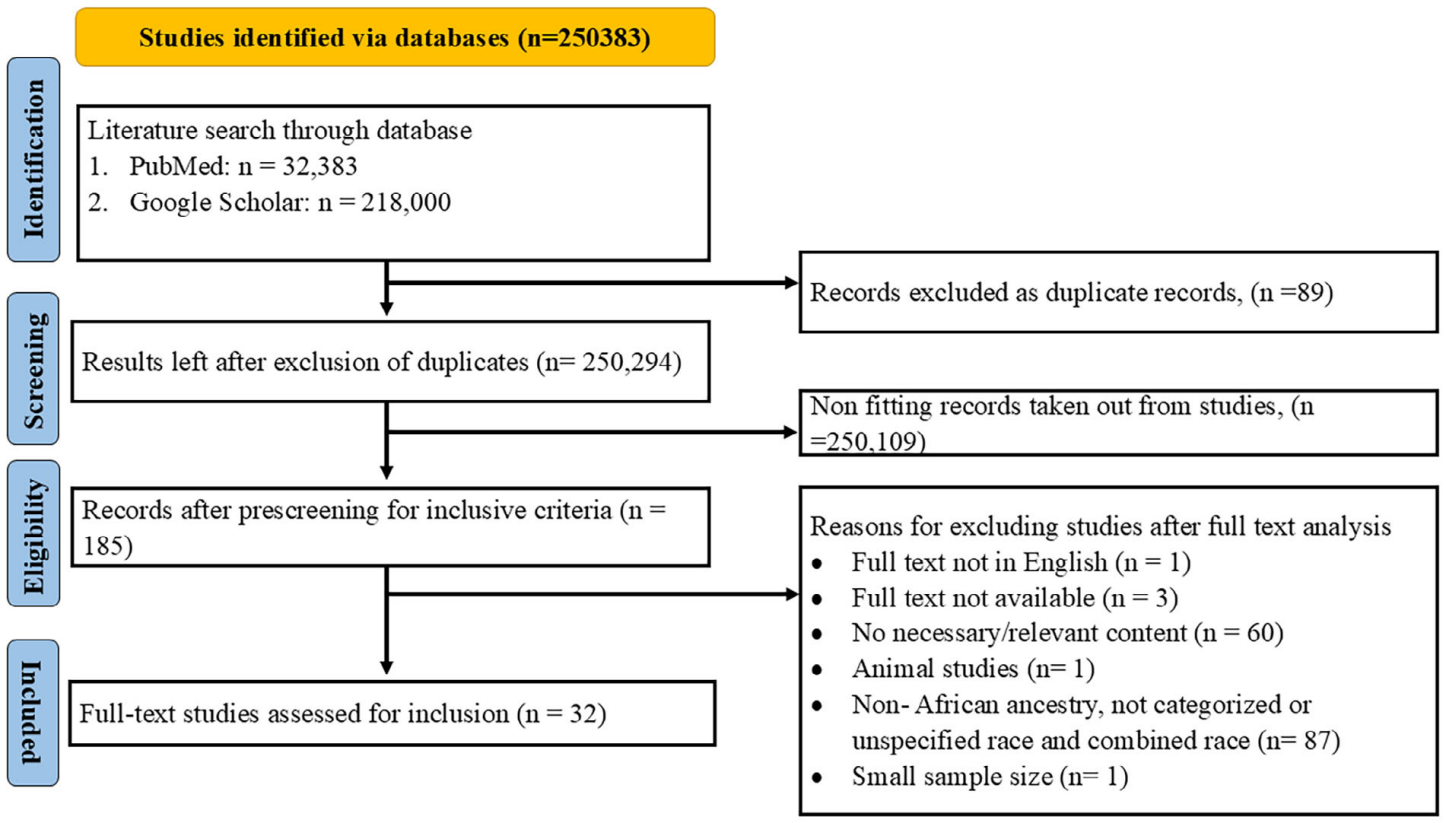
Preferred Reporting Items for Systematic Reviews and Meta‐Analyses (PRISMA) flow diagram of the screening and selection of studies.

A detailed search strategy has been included as supporting information. Two reviewers independently assessed the titles, abstracts, and full texts, resolving any discrepancies through discussion or by consulting a third reviewer. The selection process was documented in a PRISMA flow diagram, detailing reasons for exclusion at each stage (Figure 1). A standardized data extraction form was used to collect study characteristics (e.g., author, year, location, study design), population demographics, cytokine panels, and immune markers analyzed (refer to Table 1 for the tools used for their analysis), and the various neurodegenerative disease conditions (Table 1). Again, two reviewers independently extracted data, with discrepancies resolved by a third reviewer. All references identified in this systematic review were done using Mendeley software (version 1.19.8). The references were organized, checked for duplicates, and screened by categorizing studies on their eligibility status (included, excluded) with the referencing software. Advanced features in the reference manager such as automatic full‐text retrieval were engaged to annotate, track, and make decisions at each stage of the review, ensuring that there was a transparent and reproducible workflow. Risk of bias for each study selected was assessed using recommended tools, which included the Newcastle–Ottawa Scale (NOS) for cohort and case–control studies, and the Q‐Genie tool for genetic association studies. For the NOS assessment, papers were evaluated based on three key criteria: selection, comparability, and outcome/exposure. In case–control and cohort studies, a maximum of one star was awarded for each numbered item within the selection and exposure/outcome categories, while comparability was assigned a maximum of two stars. Similarly, for cross‐sectional studies, up to three stars were awarded for the selection category, with a maximum of two stars granted for comparability and outcome categories. The NOS scores for the various papers are presented in Table 1. For the Q‐Genie tool, papers were assessed across 11 categories, including study rationale, selection and definition of the outcome of interest, technical classification of exposure, and more. Each category was graded on a scale ranging from poor to excellent, ensuring a comprehensive evaluation of study quality (Table 1). Two authors independently conducted the quality assessment. Based on this evaluation, studies were included or excluded from the final review. Any uncertainties regarding inclusion or exclusion were discussed collaboratively among the two authors, with a third author available to resolve conflicts if necessary. Findings of studies were summarized using a narrative synthesis due to expected heterogeneity precluding quantitative pooling. The expected sources of heterogeneity included clinical differences (variations in participant characteristics, interventions, or outcomes), methodological differences (study design variations), and statistical differences. These factors, along with variations in risk of bias across studies, influenced the decision to adopt a narrative synthesis approach. No formal ethical approval was required because this systematic review exclusively depended on published and publicly available data.[Table alz70514-tbl-0001]

**TABLE 1 alz70514-tbl-0001:** Sample characteristics and important findings of selected studies.

Disease/conditions under study	Study design	Sample size and sample used	Population representation	Cytokine or immune marker used	Type of analytical tool or assay and data	Key findings on the role of cytokine or immune marker	Reference
AD	Prospective cohort study	*n* = 91 AAs, 868 NHW Serum/DNA	AAs and NHW	sTREM2	Non‐genetic and genetic GWAS	Findings suggest that race may be associated with risk for genetic variants of TREM2 that influence AD‐related inflammation	Schindler et al.^33^
AD	Case–control	*n* = 25 cases, 25 controls Blood plasma	African–Egypt	IL‐8	Non‐genetic ELISA	IL‐8 were significantly higher in patients with AD (*P* < 0.001) and had a significant negative effect on all cognitive assessment tests. Plasma levels of IL‐8 level could be a useful biomarker in following AD progression	Alsadany et al.^23^
CA		AAs/BI (*n* = 12) Blood plasma	AAs/BI	BDNF	Non‐genetic MSD assay	Complex attention (*r* 0.629) and processing speed (*r* 0.734) were significantly (*P* < 0.05) related to the plasma BDNF values Interventions targeting increasing BDNF to potentially improve cognition is recommended	Traylor et al.^45^
AD	Observational study	*n* = 31 AAW Blood plasma	AAW	IL‐10	Non‐genetic SAL or MAL	IL‐10 may incite inflammation, leading to impaired aspects of executive function and short‐term memory in this sample of African American women at risk for developing AD	Patel et al.^28^
CA	Cross‐sectional study	*n* = 1479 AAs, 1232 EAs Blood plasma	AAs/EAs	hCRP, IL‐6, sTNFR1 sTNFR2	Non‐genetic CRP‐ITA IL‐6, TNFR‐MA	Associations were not found between cognition and sTNFR2, CRP, or IL‐6 in EAs Adverse associations between inflammation and cognitive function were especially apparent in AAs, primarily involving markers of TNF‐α activity	Windham et al.^49^
HV(CA)	Cross‐sectional study	*n* = 514 AAs,773 NHW) Blood plasma	AAs‐NHW	CRP, IL6, sTNFR1 sTNFR2	Non‐genetic CRP‐ITA IL6, TNFR‐MA	Associations between IL‐6 or CRP and HV were not supported Higher levels of sTNFRs were associated with smaller hippocampi Longitudinal data are needed to determine whether these biomarkers may help to identify risk of late‐life cognitive impairment	Schmidt et al. (2016)
CA	Cross‐sectional study	African–Egypt (*n* = 94) Blood serum	African–Egypt	IL‐6, (ICAM‐1)	Non‐genetic IL‐6‐ACL ICAM‐ELISA	Significant impairment in attention and sensory memory was found in subjects with high IL‐6 level This could not be detected in subjects with high ICAM‐1 level High IL‐6 level in the serum is accompanied by significant impairment in attention and sensory (intentional) memory	Elwan et al.^56^
MS	Retrospective cross‐sectional study	*n* = 150 AAs, 150 W CSF	AAs‐W	Intrathecal IgG (IgG index)	Non‐genetic NPM	AA patients with MS have lower GM volume and a stronger inverse correlation between GM volume and CSF IgG index, compared to Whites These findings suggest a potentially prominent role of humoral immunity in mediating tissue injury in AA patients with MS	Seraji‐Bozorgzad et al.^41^
CA	Prospective cohort study	*n* = 132 AAs, 63 WUA Blood serum	AAs‐WUA	IL‐1β, IL‐6, IL‐8	Non‐genetic MUsA	Among Whites, unlike among African‐Americans, IL‐6 was associated with a better baseline performance on two tests of verbal and working memory Cytokines were shown to be associated with age‐related cognitive decline among middle‐aged and older urban adults in an age group and race‐specific manner	Beydoun et al. (2019a)
AD	Prospective cohort study	AAs (*n* = 19) DNA	AAs	*IGHG* and *FCGRIIB* genes	Genetic IGHG‐ rtPCR FCGRIIB‐rtPCR	Risk of developing AD associated with GM6 positivity was marginally different in non‐FCGRIIB TT participants compared to FCGRIIB TT participants In non‐FCGRIIB TT participants, the risk of developing AD was > 2‐fold higher in GM6‐positive participants compared to GM6‐negative participants (HR = 2.44) This is the first report suggesting that immunoglobulin GM allotypes might play a role in AD etiology among AAs	Pandey et al.^30^
AD	Population‐based study	*n* = 730 Nigerian African, 803 Caucasian DNA	Nigerian African and Caucasian	α1‐antichymotrypsin (ACT) gene	Genetic PCR	Higher frequencies of the ACT*A (78.6% vs. 48.4%; *P* < 0.001) and APOE*4 (25.6% vs. 15.6%; *P* < 0.001) alleles were observed in Nigerian Blacks than in Whites Sex‐specific non‐random association between the two polymorphisms was observed in Black women but this was not as strong as observed in White women	Kamboh et al.^37^
AD	Cross‐sectional Case–control study	*n* = 60 cases, 68 controls Blood plasma	South African	ESR, WBC, MC, TNF‐α, IL‐1β, TGF‐β), IL‐10, osteopontin	Non‐genetic (TNF‐α, IL‐1β, IL‐10)‐BPPA, (TGF‐β)‐MA	Mild AD participants had higher ESRs and IL‐1β levels compared to moderate AD, more severe AD and control participants The inflammatory response in AD changed with disease progression Pro‐inflammatory systemic changes were seen early in the disease but glial activation in the CNS was observed later	Grace^29^
MS	Case–control study	*n* = 66 cases, 148 controls CSF	AAs‐WAs	(IgG) index, WBC	Non‐genetic Study NTM	Measures of CSF humoral activity were all higher in the AA group. CSF humoral immune response is more active among AAs than among WAs with MS	Rinker et al.^42^
AD/MCI	Case–control study	*n* = 32 cases,9 controls Blood plasma	Algerian African	IFN‐γ and TNF‐a, NO	Non‐genetic (IFN‐γ, TNF‐α)‐ELISA NO‐SPM	High levels of NO are associated with an elevation of TNF‐α levels in severe stage of AD Data indicate that the proinflammatory cytokine production seems, in part, to be involved in neurological deleterious effects observed during the development of AD through NO pathway	Belkhelfa et al.^38^
AD	Case–control study	*n* = 32 cases, 9 controls) Brain tissue BA21	AAs‐C	IL‐1β, MIG, TRAIL, FADD, IL‐3, IL‐8, CCL25, CCL26 and CX3CL1	Non‐genetic BPPHC	IL‐1β, MIG, TRAIL, and FADD levels were significantly increased in AAs while levels of IL‐3 and IL‐8 were significantly decreased Overall levels of CCL25, CCL26, and CX3CL1 were significantly decreased in women Increased activation of NLRP3 inflammasome in AAs is consistent with the current results	Ferguson et al.^12^
CA	Cross‐sectional study	*n* = 59 AAs, 219 W Blood	AAs‐C	IL6, IL‐8 CRP, TNF‐α	Non‐genetic MPK	There were significant interactions between IL‐8 and race for the Recall a Pattern (*P *= 006) and the Digit Symbol Substitution (*P *= 014) tests as cognitive performance test Findings suggest a stronger association between IL‐8 and cognitive performance in AAs than Whites	Goldstein et al.^52^
CA	Prospective cohort study	*n* = 1255 AA, 1776 C Blood plasma	AAs‐C	IL‐6, CRP, TNF‐α	Non‐genetic ELISA	There was no significant interaction between race and inflammatory marker on cognition Serum markers of inflammation, especially IL‐6 and CRP, are prospectively associated with cognitive decline in well‐functioning elders These findings support the hypothesis that inflammation contributes to cognitive decline in the elderly	Yaffe et al.^61^
AD	Prospective cohort study	*n* = 205 AAs, 632 EAs DNA	AAs‐EAs	FCGRIIB (rs1050501 C/T) and PILRA (rs1859788 A/G) genes	Genetic rtPCR	In the AA cohort, subjects homozygous for the C allele of FCGRIIB were >4 times as likely to develop AD as those homozygous for the alternative T allele Significant associations between FCGRIIB rs1050501 genotypes and time to development of AD in AA was found, but not in the EA, cohort	Pandey et al.^31^
CA	Prospective cohort study	Black (*n* = 231), Whites (*n* = 234), Hispanic (*n* = 724), other race (*n* = 17) Blood plasma	Black, White, other, Hispanic	IL‐6, CRP	Non‐genetic ELISA	Sample with IL‐6 levels above the median had a greater rate of cognitive decline than those with levels below the median We did not find racial/ ethnic differences in the effect of IL‐6 on cognitive decline, and these results are applicable to a racially/ethnically diverse population	Economos et al.^62^
AD/CA	Prospective cohort study	AAs (case *n* = 34, control *n* = 44), White (case *n* = 40 control *n* = 75) Blood plasma	AAs‐C	IL‐9, IL‐10, IL‐8, IL‐7, TNF‐α	Non‐genetic IL‐9‐IHC (IL‐10, IL‐8, IL‐7, TNF‐α)‐ MMMAPHCP	Immunohistochemical analysis revealed glial cells immunoreactive to IL‐9, showed AD to correlate with molecular changes of IL‐9 upregulation only in AAs but not Whites Baseline and AD‐associated IL‐9 differences between AAs and Whites point to distinct molecular phenotypes for AD according to ancestry Genetic and non‐genetic factors need to be considered in future AD research involving unique populations	Wharton et al.^19^
PD	Case–control study	Case (*n* = 46) Control (*n* = 21) Blood serum	SA‐mixed ancestry	IFN‐γ, IL‐1β, IL‐6, TNF‐α, CRP	Non‐genetic (IFN‐γ, IL‐1β, IL‐6, TNF‐α)‐ MITA CRP‐CAA	PD status was associated with significant elevated levels of IL‐6 (OR = 34.97; *P* = 0.0254) and lower levels of CRP (OR = 0.57; *P* = 0.0401), after adjusting for sex The findings observed in this study contribute to the evidence that immune processes are dysregulated in NPDs; however, future studies are required to provide further insight into the relationship between pro‐inflammatory markers and NPD	Moodley^39^
AD	Prospective cohort study	ABs (case *n* = 57, control *n* = 42)αα, AW (case *n* = 718, control *n* = 511) DNA	ABs‐AW	BDNF	Genetic DPA‐PCR	No significant difference in allele, genotype, or estimated haplotype frequencies was observed between AD cases and controls within the American White and Black cohorts for the G196A and C270T BDNF polymorphisms However, the frequency of the G196A allele was significantly lower in American Black subjects compared to Whites The finding does not support any association between the BDNF/G196A or C270T polymorphism and the risk of sporadic LOAD among American Whites or Blacks	Desai et al.^36^
CA	Cross‐sectional study	AC (*n* = 290) Blood plasma	AC	IL‐6, CRP, SAA	Non‐genetic CRP‐TMA IL6‐ELISA SAA‐IPM	After adjustment for potential confounding factors, raised levels of IL‐6 (> 3.1pg/ml) were associated with cognitive decline in the total sample (OR 2.9, 95% CI 1.1–7.5), but no associations were found for CRP or SAA Raised IL‐6 but not CRP predicted cognitive decline in this population Inflammatory changes associated with cognitive decline may be specific to particular causal pathways	Jordanova et al.^57^
AD	Case–control study	AAs (case *n* = 159, control *n* = 162) Blood plasma	AAs	IL‐6, IL‐10, TNF‐α	Non‐genetic MSHCPA	Older age was associated with higher plasma A 42, tau, and TNF‐α Females had lower IL‐10 levels Inflammatory proteins had strong pairwise correlations among themselves and with A 42 Plasma inflammatory biomarkers may reflect correlated pathologies	Deniz et al.^6^
D	Case–control study	Congolese African (case *n* = 44, Control *n* = 41) Blood plasma	Congolese African	IL‐6, IL‐10, TNF‐α	Non‐genetic SHNPA	Inflammatory marker concentrations measured (IL‐1b, IL‐10, and TNF‐α), did not significantly differ by age and by sex and between those with dementia and those without These inflammatory markers did not have higher AUC compared to other non‐inflammatory plasma biomarkers	Ikanga et al.^13^
AD/VD	Case–control study	African‐ Egypt (Cases *n* = 10 VD, 10 AD*, control *n* = 20) Blood serum	African Egypt	IL‐6, CRP, Alpha‐globulins	Non‐genetic EASIA	Serum levels of IL‐6 and CRP were significantly elevated among patients with both types of dementia compared to normal elderly subjects alpha1 – and alpha 2 ‐globulins were able to discriminate between AD and VD IL‐6 levels could be used to differentiate dementia from normal aging indicating inflammation role in both types of dementia	Helmy et al.^63^
CA	Prospective cohort study	AAs (*n* = 1010)	AAs	CRP, IL‐6, (TNFR)‐1 and TNFR2	Non‐genetic CRP‐ITA (IL‐6, TNFR)‐MA	Circulating markers of CRP and IL‐6 may be differential risk factors for men and women in relation to cognitive decline A novel inflammation marker, sTNFR1, may be a useful predictor of memory decline in older adults	West et al.^60^
CA	Prospective cohort study	AAs (*n* = 1467), W (*n* = 1107) Blood serum	AAs‐W	CRP, ESR	Non‐genetic CRP‐ITA ESR‐ WPCSFKA	CRP was linked to poorer attention in older women (> 50 years, γ01 = −0.024 ± 0.007, *P* < 0.004) and AAs (γ01 = −0.029 ± 0.008, *P* < 0.001) ESR was related with poorer performance on attention tests among AAs Strong associations between systemic inflammation and longitudinal cognitive performance were detected, largely among older individuals (> 50 years) and AAs Randomized trials targeting inflammation are warranted	Beydoun et al.^59^
AD	Case–control study	AAs (cases *n* = 525, controls, *n* = 2351) DNA	AAs	sTREM2	Genetic ES‐PCR	The coding variants within the extracellular domain of TREM2 previously shown to confer LOAD risk in Whites were extremely rare in this AA cohort and did not associate with LOAD risk The findings suggest that TREM2 coding variants also confer LOAD risk in AA, but implicate variants within different regions of the gene than those identified for White subjects	Jin et al. (2015a)
CA	Longitudinal cohort study	BA (*n* = 280) Blood serum	BA	(IL‐6, IL‐10, IL‐1β), TNF‐α, CRP, IL‐1ra, VCAM, IL‐6r, MMP9	Non‐genetic ELISA	For older Black adults, chronic, but not acute, inflammation may be a risk factor for changes in cognition Higher acute inflammation associated with lower level but not change in cognition Higher chronic inflammation associated with faster decline in cognition Chronic inflammation may increase risk for cognitive decline in older Black adults	Boots et al. (2022)
AD	Prospective cohort study	AAs (*n* = 209) EA (*n* = 628) DNA	AAs‐EA	IgG1 (GM 3 and GM 17), IgG2 (GM 23+ and GM 23−), and HLA‐DRB1 rs9271192 (A/C) alleles	Genetic ‐rtPCR	There was a significant interaction: In the presence of GM 3 (i.e., GM 3/3 and GM 3/17 subjects), the presence of the HLA‐C allele was associated with a 4‐fold increase in the likelihood of developing AD compared to its absence In the absence of GM 3 (GM17/17 subjects), however, the presence of the HLA‐C allele was not associated with time to development of AD	Pandey et al.^31^
MS	Retrospective multicenter cohort study	AAs (*n* = 673), W (*n* = 717) DNA	AAs‐W	HLA‐DRB1, HLA‐DQB1	Genetic SNPs‐PCR	Role of HLA in MS not limited to disease susceptibility but genes in the locus also influence clinical outcomes	Cree et al.^43^

Abbreviations: AA, African American; AB, African Black; AC, African Caribbeans; ACL, automated chemiluminescence; AD, Alzheimer's disease; AUC, area under the curve; AW, American White; BA, Black adults; BDNF, brain‐derived neurotropic factor; BI, Black individuals; BPPA, Bio‐Plex Pro Assay; BPPHC, Bio‐Plex Pro Human Chemokine 40‐plex; C, Caucasian; CA, cognitive assessment; CAA, chemistry analyzer assay; CCL25, C‐C motif chemokine ligand 25; CCL26, C‐C motif chemokine ligand 26; CI, confidence interval; CNS, central nervous system; CRP, C‐reactive protein; CSF, cerebrospinal fluid; CX3CL1, fractalkine; DPA‐PCR, duplex pyrosequencing assay‐polymerase chain reaction; EA, European American; EASIA, enzyme‐amplified sensitivity immunoassay; ELISA, enzyme‐linked immunosorbent assay; ES, exonic sequencing; ESR, erythrocyte sedimentation rate; FADD, Fas‐associated death domain; FCGRIIB, fragment of crystallization of immunoglobulin G receptor; GM, gray matter; GWAS, genome‐wide association study; hCRP, higher sensitivity C‐reactive protein; HLA, human leukocyte antigen; HV, hippocampi volume; ICAM, intercellular adhesion molecule; IFN‐γ, interferon gamma; IgG, immunoglobulin G; IGHG, immunoglobulin heavy chain; IHC, immuno‐histochemistry; IL, interleukin; IL‐1β, Interleukin 1 beta; IL‐1ra, Interleukin 1 receptor antagonist; IL‐6r, Interleukin‐6 receptor; IPM, immunonephelometry; ITA, immunoturbidometric assays; LOAD, late‐onset Alzheimer's disease; MA, multiplex assay; MAL, multiplex assays in a Luminex 200 platform; MC, monocyte count; MCI, mild cognitive impairment; MIG, monokine induced by gamma inteferon; MITA, multiplex and immunoturbidimetric assay; MMMAPHCP, Merck‐Milliplex MAP Human Cytokine Panel; MMP, matrix metallopeptidase‐9; MPK, Multiplex Kit; MS, multiple sclerosis; MSHCPA, Multiplex Simoa Human Cytokine3‐Plex Assay; MSD‐assay, Meso Scale Discovery assay; MUsA, Multiplex Ultra‐sensitivity Assay; NHW, non‐Hispanic White; NLRP3, nod‐like receptor protein 3; NO, nitric oxide; NPD, neuropsychiatric disorder; NPM, nephelometry; NTM, no tool mentioned; OR, odds ratio; PD, Parkinson's disease; PILRA ACT, α1‐antichymotrypsin; rtPCR, real time polymerase chain reaction; SA, South African; SAA, serum amyloid A; SAL, Singleplex Assays in a Luminex 200 platform; SHNPA, Simoa Human Neurology 4‐PLEX E Assay; SNP, single nucleotide polymorphism; SPM, spectrophotometry; sTREM2, soluble triggering receptor expressed on myeloid cells 2; sTNFR1 and 2, soluble tumor necrosis factor receptor 1 and 2; TGF‐β, transforming growth factor‐beta; TMA, turbidimetric immunoassay; TNF‐α, tumor necrosis factor‐α; TRAIL, tumor necrosis factor related apoptosis‐induced ligand; VCAM, vascular cell adhesion molecule; VD, vascular dementia; W, White; WBC, white blood cell count; WPCSFKA, Westergren Photochemical Capillary Stopped Flow Kinetic Analysis; WUA, White urban adult.

## 3 RESULTS AND DISCUSSION

### 3.1 Cytokines and other immune markers in AD

Ethnicity‐related variations in AD have gained increasing attention, particularly in the context of inflammatory and genetic markers that influence disease progression. IL‐9 has been identified as a key regulator that functions to promote the activation of neuroglial cells, boosting their capacity to phagocytose and clear Aβ deposits, which helps in limiting the formation of amyloid plaques. Furthermore, IL‐9 plays a role in altering the permeability of the blood–brain barrier, potentially allowing peripheral immune cells to enter the brain and thereby modulating the local inflammatory landscape.^18^ Interestingly, glial cells reactive to IL‐9 exhibit a correlation between AD and IL‐9 upregulation in African Americans (AAs).^19^ However, this molecular change is not observed in Caucasians.^19^ Baseline and AD‐associated IL‐9 differences between AAs and Caucasians suggest distinct molecular phenotypes for AD based on ancestry. This finding is consistent with studies done on other racial populations, aside from those of African ancestry, which also reported increased serum levels of IL‐9, linking the increased expression of systemic inflammatory markers to the onset of neurodegeneration.^20^

Similarly, differences in chemokine and cytokine levels have been observed in *post mortem* brain tissue samples from the BA21 region of AAs and Caucasians with AD.^12^ Levels of IL‐1β, MIG, TRAIL, and FADD are significantly increased in AAs with AD, while levels of IL‐3 and IL‐8 are significantly decreased in same group compared to the Caucasian group.^12^ Further analysis reveals a significant elevation in IL‐1β levels, with an ≈ 109% increase observed in AAs, highlighting potential ethnicity‐related differences in inflammatory responses in AD.^12^ IL‐1β, a proinflammatory cytokine, is mainly released by activated microglia in response to the buildup of Aβ plaques and other forms of neuronal stress.^21^ Its maturation is carefully regulated by inflammasome complexes, especially the nod‐like receptor protein 3 (NLRP3) inflammasome, which converts inactive pro‐IL‐1β into its active form.^21^ Once released, IL‐1β increases the inflammatory response by stimulating the production of more cytokines, chemokines, and adhesion molecules, thereby enhancing the activation of the neuroglial cells and sustaining neuroinflammation.^21^ Moreover, IL‐1β disrupts synaptic plasticity, impedes long‐term potentiation (LTP), and promotes the hyperphosphorylation of tau proteins through the activation of signaling pathways like p38 MAPK and GSK‐3β, eventually contributing to the progression of neurofibrillary tangles.^22^

IL‐8 presents a complex role, functioning as both a neuroprotective and neurodegenerative factor. The significant decrease in IL‐8 levels observed among AAs^12^ contrasts with findings from an Egyptian population,^23^ where increased IL‐8 levels in an African population were reported to have a notable negative impact on cognitive assessment tests. Elevated levels of IL‐8 in AD have also been reported,^24^ indicating that plasma IL‐8 levels may serve as a valuable biomarker for monitoring disease progression. A comprehensive review examining the impact of IL‐8 on neurons supports these varied findings, highlighting its dual role.^25^ IL‐8 promotes neuroprotection through increased BDNF production while also contributing to neurodegeneration by enhancing apoptosis and exacerbating Aβ‐induced inflammation.^25^

Meanwhile, IL‐10 serves an essential role in controlling neuroinflammation and the formation of amyloid plaques in AD, with both genetic and functional studies underscoring its importance. Genetic polymorphisms in the IL‐10 gene promoter region have been associated with varying susceptibility to AD, where reduced IL‐10 expression is linked to increased inflammatory responses and a greater risk of disease development.^26^ Functionally, IL‐10 works by limiting the production of reactive oxygen species (ROS) in microglial cells, which in turn prevents the caspase‐1‐dependent activation of IL‐1β a critical driver of sustained neuroinflammation and subsequent neuronal damage.^27^ Additionally, higher IL‐10 levels have been observed in older individuals with AD among the AA population within a specific batch, suggesting a potential link between age and anti‐inflammatory responses in AD.^6^ Conversely, female sex is linked to lower IL‐10 levels, with the strongest effects seen in AD cases. The significant association between higher IL‐10 levels and older age in AD cases indicates that IL‐10 could be a marker influenced by age in the presence of AD. Moreover, the observed association between female sex and lower IL‐10 levels suggests potential sex‐based differences in IL‐10 regulation within this population. Furthermore, at‐risk AD female patients with elevated IL‐10 demonstrate significantly poorer performance on the Trail‐Making Test but better on the Brief Psychosocial Therapy, which assesses motor‐cognitive integration and short‐term memory.^28^ In a South African population, high baseline levels of TNF‐α, low baseline levels of IL‐10, and the presence of the *APOE* ε4 allele are independently linked to greater cognitive decline in AD.^29^ The studies emphasize the significant role of IL‐1β, IL‐9, IL‐8, and IL‐10 in AD, highlighting the influence of ancestry on disease progression. IL‐9 supports neuronal resilience, IL‐1β drives neuroinflammation, and IL‐10 regulates immune responses, revealing ancestry‐specific immune profiles that shape AD pathology in African populations. The distinct variations in these immune markers across different groups underscore the need for inclusive research to refine biomarker applications and develop targeted intervention strategies.

Beyond cytokines, genetic predisposition plays a significant role in AD susceptibility among individuals of African descent. IgG3 allotype GM6 expressed exclusively in people of African descent is recognized as associated risk of developing AD.^30^ The risk of developing AD is more than 4‐fold and 2‐fold higher in GM6‐positive participants compared to GM6‐negative participants in non‐GM17/GM17 participants and non‐FCGRIIB TT participants, respectively. This therefore suggests that immunoglobulin GM allotypes might play a role in AD etiology among people of African descent, especially AAs. Additionally, none of the GM or HLA alleles individually are associated with time to development of AD in AAs. However, in the presence of GM 3, the HLA‐C allele is associated with a 4‐fold increase in the likelihood of developing AD compared to its absence.^31^ AA subjects that are homozygous for the C allele of *FCGRIIB* *rs1050501* are more than four times as likely to develop AD as those homozygous for the alternative T allele.^32^ Significant associations have also been reported between *FCGRIIB rs1050501* genotypes and time to development of AD in the AA cohort but not in the European American (EA) cohort.^32^

The exact mechanisms by which *TREM2* variants increase the risk of neurodegenerative diseases are still being investigated, but functional implications of their genetic variations involve a combination of impaired microglial function, altered inflammation, and disrupted clearance of amyloid plaques, which in turn exacerbate and accelerate neurodegeneration.^33^ Additionally, the risk for genetic variants of *TREM2* may be associated with race and could influence AD‐related inflammation.^33^ AAs exhibited lower CSF soluble TREM2 (sTREM2) levels compared to Whites, possibly due to a higher prevalence of genetic variants linked to reduced CSF sTREM2 levels among AAs. Notably, CSF sTREM2 serves as a marker for TREM2‐mediated microglial activity, which plays a crucial role in the immune response to amyloid plaques in AD. Also, *TREM2* supports brain homeostasis and modulates inflammation in neurodegeneration and injury. Additionally, TREM2 coding variants contribute to the risk of late‐onset AD in AAs.^34^ However, the implicated variants are located in different regions of the gene in AAs compared to those found in Caucasian individuals. Two other variants of TREM2, p.R47H and p.R62H, have also been found to be significantly associated with AD risk. Gene‐based tests reveal that these TREM2 variants are significantly associated with AD at the genome‐wide level. Even when p.R47H is excluded, the association of TREM2 variants with AD remains highly significant, suggesting that additional TREM2 variants contribute to AD risk.^35^

BDNF, due to its critical role in neuronal development and survival, also offers a key functional candidate gene for AD. BDNF also serves as a neurotransmitter modulator and is essential for neuronal plasticity, which supports learning and memory processes. Again, BDNF has been shown to promote the survival of dopaminergic neurons, which are affected in PD, and also seen to play significant role in protecting striatal neurons from degeneration, which is a hallmark of Huntington's disease.^36^ However, no significant differences in allele, genotype, or estimated haplotype frequencies has been found between AD cases and controls within the American White and Black cohorts for the BDNF/G196A or C270T polymorphisms. Additionally, there have been no association between the BDNF‐G196A or C270T polymorphisms and the risk of sporadic late‐onset AD in American White or Black cohorts.^36^ On the other hand, the C270T polymorphism has been associated with lower Mini‐Mental State Examination (MMSE) scores among Blacks. The variability in outcomes observed in BDNF genetic studies may be influenced by confounding factors such as age, sex, environmental influences, sample size, and ethnicity, highlighting the need for research on more homogeneous, larger populations with improved control of these variables. The α1‐antichymotrypsin (ACT*A) alleles and ACT/AA genotype have been observed to have significant higher frequencies in Blacks compared to Caucasians.^37^ AD has been linked to immune disorders characterized by a notable increase in inflammatory cytokines and elevated production of free radicals, including nitric oxide (NO).^38^ In Algeria, elevated in vivo interferon gamma (IFN‐γ) levels were observed in AD patients at the mild stage, while TNF‐α levels were notably higher in severe cases, compared to moderate AD and mild cognitive impairment (MCI), highlighting stage‐specific variations in inflammatory responses.^38^ Additionally, NO production has been related to the increased levels of IFN‐γ and TNF‐α, in mild and severe stages of AD. In severe AD, higher NO levels correlated with elevated TNF‐α. This highlights the role of proinflammatory cytokines in driving neurological damage through the NO pathway during AD progression. Genetic differences, particularly in *TREM2* variants, appear to influence AD differently across racial groups, emphasizing the importance of population‐specific research to better understand these variations. The role of inflammatory genetic predisposition in increasing the likelihood of developing AD warrants further exploration, offering potential insights into the underlying disease pathologies.

### 3.2 Cytokines and immune marker findings and PD

Although research on biomarkers related to PD in Africa exists, few studies have explored the association between inflammatory markers and PD among individuals of African ancestry or within the African region. Evidence from a study conducted in South African mixed ancestry suggests immune process dysregulation in PD, with PD status showing significantly elevated IL‐6 levels and lower CRP levels after adjusting for sex.^39^ The elevated IL‐6 levels align with previous reports of higher IL‐6 concentrations in PD among non‐African ancestry.^1^ Consistent with its pro‐inflammatory nature, increased IL‐6 levels have also been observed in similar cases,^40^ reinforcing its potential role in PD pathology. All these findings about IL‐6 are consistent with its pro‐inflammatory nature. Further studies are recommended to provide more insight into the relationship between pro‐inflammatory markers and PD.

### 3.3 Cytokines and immune marker findings and MS

Many inflammatory cytokines in association with MS have not been studied in the African ancestry. IL‐9 has, however, been identified as a vital molecule for protecting the nervous system, with the ability to counteract inflammatory damage to synapses.^18^ Gray matter volume reflects the ability of the brain to perceive, learn, and keep memories for normal day‐to‐day activities, which assesses the cognition of a person. Lower gray matter volume and a stronger inverse correlation between gray matter volume and CSF IgG index have been indicated in AAs with MS, compared to Whites.^41^ The IgG index reflects the key role of humoral immune mediation in MS. Comparative analysis of CSF humoral activity has shown higher IgG index values in AAs than in Caucasian Americans with MS, indicating more active CSF humoral immune responses in AAs.^42^ This suggests a potentially significant role of humoral immunity in mediating tissue injury in AAs with MS.

Genetic factors influencing MS have been linked to the human leukocyte antigen‐haplotype gene (*HLA‐DRB1*) and HLA‐DQB1 alleles, with the DRB1*15 allele increasing the likelihood of typical MS.^43^ Notably, opticospinal MS patients with anti‐aquaporin 4 antibodies do not carry this allele. The HLA‐DRB1*15:01 allele is recognized as a key genetic determinant of MS onset, playing a crucial role in immune system regulation and interacting with environmental factors. This allele contributes to MS by presenting self‐antigens to CD4+ T cells, initiating an autoimmune response that damages the myelin sheath of nerves in the central nervous system. Additionally, individuals carrying this allele exhibit decreased methylation in the exon 2 region of the *HLA‐DRB1* gene, leading to heightened expression in monocytes and increased activation of autoreactive T cells that mistakenly attack the body's own cells.^44^ African ancestry at the HLA region has been associated with greater disability and an increased risk of cane dependency, while the DRB1*15 allele has been linked to earlier disease onset.^43^ These associations underscore the role of HLA in both disease susceptibility and clinical progression in MS.

CSF has been the most frequently used sample for measuring cytokines and immune markers in MS, particularly in studies emphasizing humoral immune mediation as a key factor. To achieve a more comprehensive understanding of both humoral and cellular immune responses in MS pathogenesis within populations of African ancestry, further exploration of additional samples with diverse inflammatory markers is recommended.

### 3.4 Cytokines and immune marker findings and cognitive assessment

Extensive research has explored the association among cytokines, immune markers, and cognition across diverse racial groups. Plasma levels of BDNF in AA and Blacks have been found to be significantly linked to complex attention and processing speed,^45^ reinforcing its potential role in cognitive function. Studies have consistently reported higher BDNF levels as a possible enhancer of cognitive abilities.^46^, ^47^ Additionally, BDNF has been described as a marker directly associated with the onset and progression of mnemonic symptoms characteristic of various cognitive impairments,^48^ further supporting its role in cognitive health.

The relationship between specific inflammatory biomarkers and cognitive function has been examined in AAs and EAs with common vascular risk factors. Among AAs, elevated soluble tumor necrosis factor receptor (sTNFR)2 levels were linked to poorer cognitive performance across multiple domains, including global cognition, processing speed, language, memory, and executive function.^49^ Similarly, higher sTNFR1 levels have been associated with slower processing speed and diminished executive function. Elevated CRP levels corresponded with slower processing speed, while increased IL‐6 levels were tied to impaired executive function.^49^ However, no associations were observed between cognition and CRP or IL‐6 in AAs. This review underscores the significant role of inflammation in cognitive outcomes and highlights the need for further investigation into the mechanisms underlying the associations among cytokines, immune markers, and cognitive assessment.

The pronounced adverse associations between inflammation and cognitive function among AAs have been examined, with a particular focus on markers of TNF‐α activity. While TNF‐α has demonstrated significant links to cognition in some studies,^50^, ^51^ other research has reported no similar associations among AA and Caucasian populations.^52^ Additionally, some findings indicate no significant association between CRP and cognition,^49^, ^52^ as well as no causal link between IL‐6 and cognitive function.^52^, ^53^ However, in a study of high‐functioning older adults, individuals with higher IL‐6 levels were identified as being at greater risk for incident cognitive impairment,^54^ presenting a contrast to studies that reported no causal link between IL‐6 and cognition.

The hippocampus, a brain region critical for memory and learning, serves as a key indicator of cognitive function. No associations have been reported between IL‐6 or CRP and hippocampal volume.^55^ In contrast, higher levels of sTNFRs have been linked to smaller hippocampi. Further data are needed to determine whether these biomarkers may help identify the risk of late‐life cognitive impairment. Additionally, elevated IL‐6 levels have been associated with significant impairments in attention and sensory memory, whereas no similar deficits were observed in individuals with high intercellular adhesion molecule 1 levels.^56^ Additionally, in a study adjusting for confounding factors, increased IL‐6 levels were linked to cognitive decline among African Caribbean individuals, while no associations were found for CRP or serum amyloid A (SAA),^57^ suggesting that IL‐6 may be a more relevant predictor of cognitive decline in this population.

Research examining systemic inflammation and cognitive performance among AAs and White urban adults has indicated that IL‐6 is not associated with better baseline performance in verbal and working memory tests among AAs compared to Whites.^58^ Further analysis revealed that AAs had higher baseline IL‐6 levels and greater variability. Additionally, cytokines have been linked to age‐related cognitive decline in a race‐specific manner. Elevated levels of cytokines, including IL‐1β, IL‐6, and IL‐18, have also shown strong associations with cognitive performance, suggesting that increased systemic inflammation is generally correlated with poorer outcomes and accelerated cognitive decline.^58^ Faster cognitive decline has been associated with higher chronic inflammation, while higher acute inflammation has shown no notable changes in cognition.^59^ Among older Black adults, chronic inflammation has been identified as a risk factor for cognitive changes.^49^

Strong associations have been observed between systemic inflammation and longitudinal cognitive performance, particularly among older individuals (> 50 years) and AAs.^59^ CRP has been linked to poorer attention and baseline mental status, while higher erythrocyte sedimentation rate levels have been associated with diminished attention, reduced executive function, and faster verbal memory decline.^59^ Circulating markers of CRP and IL‐6 have been identified as differential risk factors for cognitive decline among AA men and women.^60^ A novel inflammation marker, sTNFR1, has also been identified as a potential predictor of memory decline in older adults.^60^ These findings highlight the significant impact of inflammation on cognitive outcomes and underscore the need for targeted interventions. Furthermore, future research on cognitive decline should incorporate a comprehensive examination of sex and racial/ethnic variations to better understand these complex interactions.

Reports on IL‐8 and its interaction with race in cognitive performance tests have shown inconsistencies. While some findings indicate a stronger association between IL‐8 and cognitive performance in AAs compared to Caucasians,^52^ other studies have found no link between race and inflammatory markers in relation to cognition. Serum markers of inflammation, particularly IL‐6 and CRP, have been identified as predictors of cognitive decline in well‐functioning older adults, supporting the hypothesis that inflammation contributes to cognitive deterioration in aging populations. However, no significant interaction has been observed between race and inflammatory markers in cognitive decline.^61^ Additionally, population‐based research among Hispanic and Blacks has shown that those with IL‐6 levels above the median experienced a greater rate of cognitive decline than those with lower levels, with no observed racial or ethnic differences in the effect of IL‐6 on cognitive decline.^62^

### 3.5 Cytokines and immune markers and dementia

Few studies have examined the association among cytokines, immune markers, and dementia among individuals of African ancestry. Research involving demented and non‐demented Congolese Africans has shown no significant differences in inflammatory marker concentrations, including IL‐1β, IL‐10, and TNF‐α, across age and sex.^13^ When assessed for diagnostic performance, these inflammatory markers did not demonstrate higher predictive accuracy compared to other non‐inflammatory plasma biomarkers for dementia. Additionally, serum levels of IL‐6 and C‐reactive protein have been found to be significantly elevated in Egyptian patients with both AD and vascular dementia (VD) compared to normal elderly individuals.^63^ While alpha‐globulins have been useful in distinguishing between AD and VD, IL‐6 levels may serve as a differentiator between dementia and normal aging, reinforcing the role of inflammation in both types of dementia. Further research on cytokines and dementia among individuals of African ancestry, particularly within the African region, is necessary to uncover potential associations that could inform therapeutic strategies and improve patient outcomes.

### 3.6 Novel findings on cytokines and immune markers in relation to various conditions

Among the inflammatory cytokines reviewed in relation to cognition across racial groups, IL‐6 has received considerable focus. Findings on IL‐6 and its association with inflammation and race in cognitive function have been mixed, with some studies reporting significant interactions while others found no association. These inconsistencies may be influenced by factors such as environmental, genetic, and lifestyle variations, as well as differences in study design and target populations. Although some studies found no association between race and IL‐6 in cognitive function, the overall findings align with established research linking IL‐6 to cognitive decline. Additionally, IL‐6 has been correlated with AD, PD, and dementia, reinforcing its role as a pro‐inflammatory marker in neurodegenerative diseases, including those affecting individuals of African ancestry.

sTNFR1, plasma levels of BDNF, and IL‐8 have been identified as risk markers for cognitive impairment in studies examining their association with cognition among individuals of African ancestry, despite limited research on these biomarkers. Further studies, particularly within the African region, are recommended to better understand their roles and effects on cognition. Additionally, across various studies, IL‐8 has been consistently correlated with neurodegeneration among individuals of African ancestry, reinforcing its significant role in disease pathology.

New insights into the genetic predisposition of inflammatory markers for AD have emerged from studies on African ancestry. Research on the IgG index (GM3, GM6) as a humoral mediation response in neurodegeneration and cognition has revealed significant interactions. The presence of the HLA‐C allele alongside GM3 has been associated with a 4‐fold increase in the likelihood of developing AD. Additionally, the risk of developing AD linked to GM6 positivity differs between non‐FCGRIIB TT participants and FCGRIIB TT participants, with non‐FCGRIIB TT individuals experiencing more than twice the likelihood of developing AD when GM6 positive compared to GM6 negative. Significant associations have also been identified between FCGRIIB rs1050501 genotypes and time to AD development in AA cohorts, but not in EA cohorts. Furthermore, genetic variants of TREM2 have been found to influence AD‐related inflammation, with TREM2 coding variants being linked to an increased risk of late‐onset AD in African ancestry. These findings highlight the potential for further research into the genetic predisposition of inflammatory markers in neurodegenerative diseases and cognitive function.

This review highlights important clinical implications for populations of African ancestry, particularly in the diagnosis, prognosis, and treatment of neurodegenerative diseases. The identified inflammatory markers offer potential as cost‐effective, less‐invasive screening tools for early detection of individuals at risk for cognitive decline and neurodegenerative disorders. Additionally, these findings support the exploration of therapeutic strategies aimed at modulating inflammation, particularly during the early stages of AD, PD, and MS. Targeted interventions, such as IL‐6 inhibition or IL‐10 enhancement, could help mitigate neuroinflammation and slow disease progression.

Future research should prioritize long‐term observational studies to better understand the role of inflammation in cognitive decline and neurodegeneration among diverse African populations. Expanding studies with larger sample sizes and more standardized methods for measuring biomarkers will further refine diagnostic criteria and treatment strategies. Ultimately, integrating these findings into clinical practice can help address health disparities by informing culturally and genetically tailored approaches to neurodegenerative disease management within African and African ancestry populations.

Despite using a comprehensive search across multiple databases, relevant studies indexed in less accessible or regional databases may have been missed. The selection criteria emphasized peer‐reviewed articles published in English, which may have led to the exclusion of potentially valuable studies in other languages. This introduces a risk of language bias, especially considering that studies conducted in African countries may be published in other languages.

## 4 CONCLUSION

This review highlights the complex interplay among inflammatory cytokines, immune markers, and neurodegenerative diseases, particularly AD, PD, MS, and cognitive decline among populations of African ancestry. The evidence suggests significant ethnic variations in the association between these biomarkers and cognitive outcomes, emphasizing the need for population‐specific research. Key findings indicate that elevated levels of inflammatory markers such as IL‐6, IL‐1β, and sTNFRs are linked to cognitive impairment and neurodegeneration, while the role of IL‐10 and IL‐8 present varying associations based on demographic factors. Studies suggest that genetic predispositions such as *TREM2* variants and immunoglobulin GM allotypes may further influence the risk of developing these conditions in individuals of African descent.

Despite some inconsistencies in the findings, particularly regarding the impact of race on inflammation and cognition, the overall trend highlights the importance of inflammation in the pathogenesis of neurodegenerative diseases. Long‐term observational research involving inflammation and the progression of cognitive decline and neurodegeneration should be conducted among participants from various ethnic groups within Africa, as this will help identify population‐specific patterns and disparities. By elucidating these relationships, we can inform diagnostic and therapeutic strategies to improve outcomes for affected individuals of African descent.

## AUTHOR CONTRIBUTIONS

*Conceptualization*: Maxwell Hubert Antwi, George Nkrumah Osei, Ansumana Bockarie, and David Larbi Simpong. *Methodology and investigation*: Maxwell Hubert Antwi, Ansumana Bockarie, George Nkrumah Osei, and David Mawutor Donkor. *Supervision*: David Larbi Simpong. *Writing—original draft*: Maxwell Hubert Antwi, George Nkrumah Osei, David Mawutor Donkor, and David Larbi Simpong. All authors revised the manuscript critically for important intellectual content. All authors read and agreed with the final manuscript.

## CONFLICT OF INTEREST STATEMENT

The authors declare no conflicts of interest. Author disclosures are available in the supporting information.

## Supporting information



Supporting Information

Supporting Information

## Data Availability

No data were used for the research described in the article.
