# Supplementary material for: Cytokines and immune biomarkers in neurodegeneration and cognitive function: A systematic review among individuals of African ancestry
Source: Alzheimers Dement. 2025 Jul 25;21(7):e70514. doi: 10.1002/alz.70514 (PMC12290485; doi:10.1002/alz.70514)
Supplement: Supplementary file 2 — Supporting Information [file ALZ-21-e70514-s001.docx]

**Search Strategy Terms**

PubMed

1. ("Cytokines" OR "Immune Biomarkers") AND ("Neurodegeneration" OR "Cognitive Function" OR "Neuroinflammation" OR "Neurodegenerative disease" OR "Neurodegenerative disease") OR ("Africans" OR "African Americans" OR "African ancestry") NOT (Review)

Google Scholar

1. ("Cytokines" OR "Immune Biomarkers") AND ("Neurodegeneration" OR "Cognitive Function" OR "Neuroinflammation" OR "Neurodegenerative disease" OR "Neurodegenerative disease") OR ("Africans" OR "African Americans" OR "African ancestry") NOT (Review)
